# Combined hepatocellular and cholangiocarcinoma originating from the same clone: a pathomolecular evidence-based study

**DOI:** 10.1186/s40880-016-0146-7

**Published:** 2016-08-24

**Authors:** Qian Zhao, Wen-Long Yu, Xin-Yuan Lu, Hui Dong, Yi-Jin Gu, Xia Sheng, Wen-Ming Cong, Meng-Chao Wu

**Affiliations:** 1 Department of Pathology, Eastern Hepatobiliary Surgery Hospital, Second Military Medical University, Shanghai, 200438 P. R. China; 2 Department of Biliary Surgery, Eastern Hepatobiliary Surgery Hospital, Second Military Medical University, Shanghai, 200438 P. R. China; 3 Department of Hepatic Surgery, Eastern Hepatobiliary Surgery Hospital, Second Military Medical University, Shanghai, 200438 P. R. China

**Keywords:** Combined hepatocellular and cholangiocarcinoma, Hepatocellular carcinoma, Intrahepatic cholangiocellular carcinoma, Loss of heterozygosity, Clonal origin, Prognosis

## Abstract

**Background:**

Combined hepatocellular and cholangiocarcinoma (CHC) is a unique subtype of liver cancer comprising both hepatocellular carcinoma (HCC) and intrahepatic cholangiocarcinoma (ICC); however, its cellular origin remains unclear. The purpose of this study was to investigate the clinicopathologic features and the clonal relationship between HCC and ICC in 34 patients with CHC.

**Methods:**

The clinicopathologic features and prognosis of the 34 CHC patients were compared with those of 29 patients with separated HCC and ICC (SHC). Loss of heterozygosity (LOH) at 10 highly polymorphic microsatellite markers was detected in 16 CHC and 10 SHC tissues for determination of the clonal origin of CHC. Expression of hepatocyte markers [hepatocyte paraffin 1 (Hep Par 1) and glypican 3 (GPC3)] and cholangiocyte markers [cytokeratin (CK)7 and 19] in tumor tissues was examined by immuno histochemical analysis.

**Results:**

In the 16 CHC specimens, the difference in LOH patterns between HCC and ICC was less than 30%, suggesting the same clonal origin of HCC and ICC. Consistent with this finding, immunohistochemical analysis revealed that hepatocyte markers (Hep Par 1 and GPC3) and cholangiocyte markers (CK7 and CK19) were simultaneously expressed in both the HCC and ICC components in 52.9% of CHC specimens, suggesting that the two components shared a similar phenotype with hepatic progenitor cells (HPCs). On the contrary, in all 10 SHC cases, the difference in LOH patterns between the HCC and ICC components was greater than 30%, suggesting different clonal origins of HCC and ICC. Overall survival and disease-free survival were shorter for patients with CHC than for patients with SHC (*P* < 0.05).

**Conclusions:**

Our results suggest that the HCC and ICC components of CHC may originate from the same clone, having the potential for dual-directional differentiation similar to HPCs. CHC tended to exhibit the biological behaviors of both HCC and ICC, which may enhance the infiltrative capacity of tumor cells, leading to poor clinical outcomes for patients with CHC.

## Background

Combined hepatocellular and cholangiocarcinoma (CHC), which is characterized by hepatocellular and biliary epithelial differentiation within the same tumor [1–3], is a unique type of primary hepatic carcinoma that is distinct from hepatocellular carcinoma (HCC) and intrahepatic cholangiocarcinoma (ICC). CHC has been reported to account for 0.4% to 14.2% of primary hepatic carcinoma cases [4]. Between January 1982 and December 2009, of 31,000 cases of primary hepatic carcinoma treated at the Eastern Hepatobiliary Surgery Hospital (EHBH) in Shanghai, China, 92.3%, 6.7%, and 1.9% were classified as HCC, ICC, and CHC, respectively [5].

Although some clinical features of CHC are similar to those of HCC and ICC [4], the biological behavior of CHC and the prognosis for patients with CHC are completely different. Because CHC has components of both HCC and ICC, the effects of its two different components on clonal origin and clinical outcomes have become a focus of attention. However, molecular alterations in CHC have not been extensively studied, and the clonal link between the HCC and ICC components of CHC remains unclear [4, 6–14].

An accurate method to identify the origin of CHC is to determine whether the tumor components (i.e., HCC and ICC) are from a single clonal origin or multiple clonal origins. For clonal analysis of CHC, the most precise techniques rely on the detection of common patterns in DNA aberrations within each tumor component (i.e., the hepatocyte- and the biliary tubular-differentiated zones). Previous studies have used the integration pattern of hepatitis B virus (HBV) DNA detection, the X-chromosome inactivation assay, and comparative genomic hybridization as methods of tumor clonal origin determination [6]. However, these methods have limitations, including only being suitable for female patients and requiring fresh tissue specimens and expensive equipment and reagents. In general, these methods are not appropriate for routine clinical pathologic analysis using paraffin-embedded blocks. Loss of heterozygosity (LOH) is a non-random allelic loss of specific chromosomal loci, and it is closely associated with silencing of known or unknown tumor suppressor genes. Our previous research indicated that LOH detection has technical advantages for clonal discrimination in recurrent HCC [15] and hepatic carcinosarcoma [16].

Although there have been several studies of CHC, systematic studies in large cohorts of CHC patients, with detailed information on diagnostic criteria, surgicopathologic features, clonal origin, and clinical prognosis, are lacking. In the present study, we analyzed the characteristics of molecular clones in 34 CHC cases and compared them with 29 cases of separated HCC and ICC (SHC) with their respective differentiations in the same liver. In this study, SHC refers to separated HCC and ICC nodules in the same liver. In addition, we retrospectively evaluated the clinicopathologic characteristics and prognostic factors related to overall survival (OS) and disease-free survival (DFS) in patients with CHC and compared them with those in patients with SHC, pure HCC, and pure ICC who underwent resection during the same period.

## Patients and methods

### Patient inclusion

The medical records of 34 patients with CHC who underwent radical surgical resection with a negative resection margin between January 1982 and December 2009 were retrieved from the database of the Department of Pathology at EHBH (Shanghai, China). The control group comprised 29 patients with SHC (58 tumor nodules) as the primary prototype model of collision tumors, 50 patients with pure HCC, and 50 patients with pure ICC who underwent radical surgical operation at EHBH during the same period. Written informed consent was obtained from all patients, and the study protocol was approved by the EHBH Research Ethics Committee.

### Detection of microsatellite LOH

Ten high-frequency HCC microsatellite LOH sites on chromosomes 4, 8, 16, and 17 were detected by using a microdissection-based, polymerase chain reaction, single-strand conformation polymorphism assay. These sites were selected because they had been described in the literature [15] and confirmed by our laboratory [16] (Table 1). An informative case (heterozygosity) was defined as a specimen having four bands of the same density in two allele genes, which appeared on gel electrophoresis of the paracancerous tissue. Assessment of CHC microsatellite LOHs was performed only for informative cases. LOH was defined as the loss of one or more allele bands in the tumor DNA or a reduction in density of >50% compared with the paracancerous tissue. Retention of heterozygosity was defined as no LOH on the tumor allele. A non-informative case was defined as a specimen with loss of only two bands on gel electrophoresis of the paracancerous tissue.

**Table 1:** Primers and characteristics of microsatellite markers

Locus	Location	Primer sequences	Size (bp)
D4S402	4q26	5q-CTTACTGTGTTGCCCAAGGT-3T 3T-AGCTCTATGATTCATTTCAAGTTTG-5G	287–323
D4S406	4q26	5q-CTGGTTTTAAGGCATGTTTG-3T 3T-TCCTCAGGGAGGTCTAATCA-5C	234–258
D8S258	8q22	5q-CTGCCAGGAATCAACTGAG-3T 3T-TTGACAGGGACCCACG-5T	144–154
D8S264	8q23	5q-ACATCTGCGTCGTCTTCATA-3C 3C-CCAACACCTGAGTCAGCATA-5C	121–145
D8S277	8p23	5p-CCAGGTGAGTTTATCAATTCCTGAG-3C 3C-TGAGAGGTCTGAGTGACATCCG-5G	148–180
D8S520	8p23	5p-CTGAAGAGCAAATGGCCCT-3T 3T-TAAGATCACATGGCCCCCT-5A	179–199
D16S514	16q21	56-CTATCCACTCACTTTCCAGG-3T 3T-TCCCACTGATCATCTTCTC-5C	117–133
D16S505	16q24.1	56-GACTGTGTCTGCCCAA-3A 3A-TCTGCCTCCATACGTG-5C	239–261
D17S831	17pter-pter	57-CGCCTTTCCTCATACTCCAG-3G 3G-GCCAGACGGGACTTGAATTA-5C	106–128
D17S938	17pter-pter	57-CCGGATTGCTACACCTAAAT-3C 3C-AACAGTCTCTNCTGGAGCAG-5A	238–258

### Evaluation of results

Based on our previous studies, the type of clonal origin of CHC was categorized as follows [6, 15–17]: single clonal origin if the difference was <30% (number of different LOH loci/number of informative loci × 100%) between the HCC and the ICC component, and multiple clonal origins if the difference was ≥30%. The tumor type of multiple clonal origins was also selected for tumors with retention of heterozygosity in at least two microsatellite markers at two different chromosomal loci and another tumor with LOH, or the detection of at least two different LOH bands in at least two different chromosomal loci between HCC and any ICC.

### Immunohistochemical analysis and semi-quantitative evaluation

Representative 4-μm serial sections were prepared from 10% formalin-fixed, paraffin-embedded tissue blocks. Immunohistochemical staining was performed on serial deparaffinized tissue sections with the Dako EnVision system (Dako Japan Co. Ltd, Kyoto, Japan). In brief, all slides were exposed to 3% hydrogen peroxide for 10 min to block endogenous peroxidase activity. Microwave antigen retrieval was performed in citrate buffer (pH 6.0) for 5 min to enhance immunoreactivity. The four primary antibodies were hepatocyte paraffin 1 (Hep Par 1; cytoplasm of hepatocytes, 1:50, Dako; Glostrup, Denmark), glypican 3 (GPC3; cytoplasm of hepatocytes, 1:200; Bio-Mosaics, Burlington, VT, USA), cytokeratin (CK)19 (cytoplasm of cholangiocytes, 1:50; Dako), and CK7 (cytoplasm of cholangiocytes, 1:50; Dako). All reagents and machines come from the Department of Pathology at EHBH.

Sections were incubated with primary antibodies in a humid chamber at 4 °C overnight, followed by incubation with anti-mouse peroxidase-conjugated EnVision antibodies at 37 °C for 30 min. Immunoreactions were visualized with 3,3-diaminobenzidine as the chromogen for 5 min at room temperature, followed by light counterstaining with hematoxylin. For a negative control, sections were incubated with Tris-buffered saline instead of primary antibodies. The immunohistochemical slides were studied by two pathologists (Cong WM and Xin YL) who were blinded to the clinical and follow-up data. The intensity of immunoreactivity was semi-quantitatively graded according to the number of positive cells, as follows: − (negative), <15% of positive cells; + (positive), ≥15% of positive cells.

### Statistical analysis

The clinicopathologic parameters of the CHC and SHC groups were compared by using the Chi square test, Fisher’s exact test, or Student’s *t* test. Clinical outcomes such as OS and DFS were assessed by using Kaplan–Meier curves. OS was defined as the interval between the date of surgery and the date of death. DFS was defined as the interval between surgery and local relapse, distant metastasis, or death, whichever occurred first. The OS and DFS are presented as median followed by range in the parentheses and mean ± standard deviation. Data analysis was performed with the SPSS software for Windows (version 13.0; SPSS Inc., Chicago, IL, USA). *P* values less than 0.05 were considered statistically significant.

## Results

### Clinicopathologic characteristics

A comparison of the clinicopathologic characteristics of patients with CHC, SHC, pure HCC, and pure ICC is shown in Table 2. The male-to-female ratios for CHC, SHC, pure HCC, and pure ICC groups were 7.5:1, 6.3:1, 15.6:1, and 2.3:1, respectively, with men being the predominant sex in all groups. Positive rate of HBV was similar in the CHC group (94.1%) and the SHC group (93.1%) and was more common in the CHC group than in the pure HCC group (86.0%, *P* = 0.301) and pure ICC group (56.0%, *P* < 0.001). In the CHC group, the frequency of cirrhosis was higher than that in the pure ICC group (58.9% vs. 24.0%, *P* < 0.001); the frequency of vascular invasion was higher than that in the pure HCC group and the pure ICC group (50.0% vs. 2.0% and 11.0%, respectively, *P* < 0.001 for both comparisons); and the frequency of lymph node metastasis was higher than that in the pure HCC group (17.6% vs. 0.0%, *P* < 0.001). Positive rate of α-fetoprotein (AFP) in the CHC group (70.6%) was similar to that in the SHC group (69.0%, *P* = 0.889) but higher than that in the pure ICC group (30.0%, *P* < 0.001). Positive rate of serum carbohydrate antigen 19-9 (CA19-9) in the CHC group (41.2%) was lower than that in the SHC group (93.1%, *P* < 0.001) but higher than that in the ICC group (2.0%, *P* < 0.001). Being positive for both AFP and serum CA19-9 were more common in the CHC group (29.4% [10 of 34]) than in the pure HCC group (2.0% [1 of 50], *P* < 0.001) and the pure ICC group (10.0% [5 of 50], *P* = 0.023). Histologic grades III–IV HCC components were more common in the CHC group than in the SHC group (88.2% vs. 62.1%, *P* = 0.015). Poor differentiation of ICC components was more common in the CHC group than in the SHC group (29.4% vs. 13.8%, *P* = 0.137).

**Table 2:** Comparison of clinicopathologic features among patients with CHC, SHC, pure HCC, and pure ICC

Characteristic	CHC (*n* = 34)	SHC (*n* = 29)	Pure HCC (*n* = 50)	Pure ICC (*n* = 50)	*P* value^c^		
					*V1*	*V2*	*V3*
Sex							
Men	30 (88.2)	25 (86.2)	47 (94.0)	35 (70.0)	0.810	0.383	0.051
Women	4 (11.8)	4 (13.8)	3 (6.0)	15 (30.0)			
Age (years)^a^	52 (24–78)	49 (24–81)	53 (26–73)	60 (42–81)	0.434	0.713	0.342
Tumor size (cm)^b^	4.05 ± 2.45	3.49 ± 2.11^▲^	6.26 ± 2.81	5.24 ± 3.42	0.237^▲^	0.146	
		2.96 ± 1.68^▼^			0.168^▼^		0.293
HBsAg (+)	32 (94.1)	27 (93.1)	43 (86.0)	28 (56.0)	1.000	0.301	<0.001
Liver cirrhosis	20 (58.9)	12 (41.4)	34 (86.0)	12 (24.0)	0.167	0.389	<0.001
Vascular invasion	17 (50.0)	9 (31.0)	1 (2.0)	6 (12.0)	0.128	<0.001	<0.001
Lymph node metastasis	6 (17.6)	4 (13.8)	0 (0.0)	10 (20.0)	0.741	0.003	0.787
AFP ≥20 μg/L	24 (70.6)	20 (69.0)	33 (66.0)	15 (30.0)	0.889	0.659	<0.001
CA19–9 ≥38 U/mL	14 (41.2)	27 (93.1)	2 (2.0)	22 (44.0)	<0.001	<0.001	0.797
AFP ≥20 μg/L and CA19–9 ≥38 U/mL	10 (29.4)	4 (13.8)	1 (2.0)	5 (10.0)	0.137	<0.001	0.023
HCC							
Histologic type							
Coarse trabecular pattern	21 (61.8)	19 (65.5)	34 (68.0)		0.025	0.834	
Fine trabecular pattern	3 (8.8)	8 (27.6)	4 (8.0)				
Others	10 (29.4)	2 (6.9)	12 (24.0)				
Edmondson grade							
I–II	4 (11.8)	11 (37.9)	9 (18.0)		0.015	0.438	
III–IV	30 (88.2)	18 (62.1)	41 (82.0)				
ICC							
Histologic type							
Tubular pattern	29 (85.3)	28 (96.6)		46 (92.0)	0.139		0.266
Others	5 (14.7)	1 (3.4)		4 (8.0)			
Differentiation grade							
Moderate and well	24 (70.6)	25 (86.2)		29 (58.0)	0.137		0.241
Poor	10 (29.4)	4 (13.8)		21 (42.0)			
Overall survival time (months)^b^	15.37 ± 2.04	24 ± 4.23	29.2 ± 4.1	10.1 ± 2.3	0.047	0.021	0.017
Disease-free survival time (months)^b^	8.85 ± 1.3	16.9 ± 3.6	22.7 ± 3.9	5.62 ± 0.7	0.038	<0.001	0.397

*CHC* combined hepatocellular and cholangiocarcinoma, *SHC* separated hepatocellular carcinoma and intrahepatic cholangiocarcinoma, *HCC* hepatocellular carcinoma, *ICC* intrahepatic cholangiocarcinoma, *HBsAg* 15 hepatitis B surface antigen, *AFP* ɑ-fetoprotein, *CA19-9* carbohydrate antigen 19-9, ▲ HCC, ▼ ICC

^a^These values are presented as median followed by range in the parentheses

^b^These data are presented as mean ±standard deviation; other values are presented as number of patients followed by percentage in the parentheses

^c^ *P* value for *V1* CHC versus SHC; *V2* CHC versus pure HCC; *V3* CHC versus pure ICC

### Clonal analysis based on LOH

For the 16 cases of CHC, the percentages of the number of different LOH microsatellite loci in the number of informative sites in HCC nodules and those in ICC nodules ranged from 22.2% to 28.5% (median, 25%). Thus, all 16 cases of CHC had a percentage of different LOH microsatellite loci in informative sites lower than 30% (100%; Table 3).

**Table 3:** LOH profiles in 16 patients with CHC

Case no.	Tumor component	LOH locus										Different LOH	Informative loci	Different LOH/informative loci (%)	Clonality diagnosis
		D4S402	D4S406	D17S831	D16S505	D17S938	D8S277	D16S514	D8S520	D8S258	D8S264				
1	T1	○	○	○	●	NI	○	●	F	●	●	2	8	25.0	SO
	T2	○	●	○	●	F	○	●	●	○	●				
2	T1	●	○	○	NI	●	●	●	○	F	○	2	7	28.5	SO
	T2	●	○	●	○	NI	●	●	●	F	○				
3	T1	○	○	●	●	○	●	●	●	●	○	2	8	25.0	SO
	T2	F	F	●	●	○	○	●	○	●	○				
4	T1	○	○	●	○	○	○	F	●	●	○	2	7	28.5	SO
	T2	NI	○	●	○	●	F	NI	●	○	○				
5	T1	●	●	○	○	●	●	○	●	○	●	2	9	22.2	SO
	T2	F	●	○	●	●	▲	○	●	○	●				
6	T1	NI	○	○	○	●	○	○	○	●	F	2	8	25.0	SO
	T2	○	○	●	○	●	●	○	○	●	○				
7	T1	F	○	NI	●	●	○	●	○	○	○	2	7	28.5	SO
	T2	●	○	●	F	○	○	●	○	○	●				
8	T1	●	○	○	●	●	F	○	●	○	○	2	8	25.0	SO
	T2	○	○	○	NI	●	○	○	●	●	○				
9	T1	○	○	●	○	NI	●	○	●	●	○	2	9	22.2	SO
	T2	●	○	●	●	●	●	○	●	●	○				
10	T1	●	○	F	●	●	○	●	○	○	●	2	8	25.0	SO
	T2	NI	○	●	●	○	○	●	●	○	●				
11	T1	○	●	○	●	NI	○	○	F	●	●	2	8	25.0	SO
	T2	○	●	○	●	F	○	●	●	○	●				
12	T1	●	○	○	○	●	●	●	○	F	●	2	9	22.2	SO
	T2	●	○	●	○	○	●	●	○	F	●				
13	T1	○	○	●	F	○	●	●	●	●	○	2	9	22.2	SO
	T2	○	●	●	●	○	●	●	○	●	○				
14	T1	○	○	●	○	▲	○	○	●	●	○	2	8	25.0	SO
	T2	○	○	F	○	●	○	○	●	○	NI				
15	T1	●	●	○	○	NI	●	○	●	●	○	2	8	25.0	SO
	T2	○	●	○	●	●	F	○	●	●	○				
16	T1	F	○	●	○	○	○	○	●	●	○	2	8	25.0	SO
	T2	○	○	●	○	●	NI	○	●	○	○				

*LOH* loss of heterozygosity, *CHC* combined hepatocellular and cholangiocarcinoma, *T1* HCC component of CHC, *T2* ICC component of CHC, ● LOH, ▲ missing but not in the same band, ○ retain, *NI* non-informative, *F* not done, *SO* single clonal origin, *MO* multiple clonal origins

For the 10 cases of SHC, the percentage of different LOH microsatellite loci in informative sites in HCC nodules and those in ICC nodules ranged from 33.3% to 42.8% (median, 37.5%). All 10 cases had a percentage of different LOH microsatellite loci in informative sites higher than 30% (100%; Table 4).

**Table 4:** LOH profiles in 10 patients with SHC in the same liver

Case no.	Tumor nodule	LOH locus										Different LOH	Informative loci	Different LOH/informative loci (%)	Clonality diagnosis
		D4S402	D4S406	D17S831	D16S505	D17S938	D8S277	D16S514	D8S520	D8S258	D8S264				
1	T1	○	○	○	○	NI	○	●	●	●	○	3	8	37.5	MO
	T2	●	●	○	○	●	○	F	●	○	○				
2	T1	●	NI	○	○	●	●	●	○	NI	○	3	8	37.5	MO
	T2	○	NI	○	○	○	●	●	○	F	●				
3	T1	○	○	●	○	F	●	●	●	●	○	3	9	33.3	MO
	T2	○	○	●	●	F	○	●	○	●	○				
4	T1	○	●	●	○	○	○	○	●	●	F	3	8	37.5	MO
	T2	○	○	●	○	●	F	○	●	▲	○				
5	T1	●	●	○	○	●	●	●	F	●	○	3	9	33.3	MO
	T2	●	●	○	●	●	○	●	F	●	●				
6	T1	●	○	○	F	●	○	●	○	●	F	3	8	37.5	MO
	T2	●	○	●	F	●	●	○	○	●	NI				
7	T1	●	○	○	●	●	F	●	F	○	○	3	8	37.5	MO
	T2	●	○	●	●	○	F	●	F	○	●				
8	T1	○	○	NI	●	●	NI	▲	●	●	F	3	7	42.8	MO
	T2	●	○	NI	○	●	NI	●	●	●	NI				
9	T1	○	○	●	○	F	●	●	●	●	○	3	9	33.3	MO
	T2	○	○	●	●	F	○	●	○	●	○				
10	T1	●	○	●	○	○	○	●	F	○	○	3	8	37.5	MO
	T2	●	F	●	●	○	●	●	○	○	●				

*SHC* separated hepatocellular carcinoma and intrahepatic cholangiocarcinoma, *T1* HCC nodule of SHC, *T2* ICC nodule of SHC, ● LOH, ▲ missing but not in the same band, ○ retain, *NI* non-informative, *F* not done, *SO* single clonal origin, *MO* multiple clonal origins

### Immunohistochemical findings

**Fig. 1:**
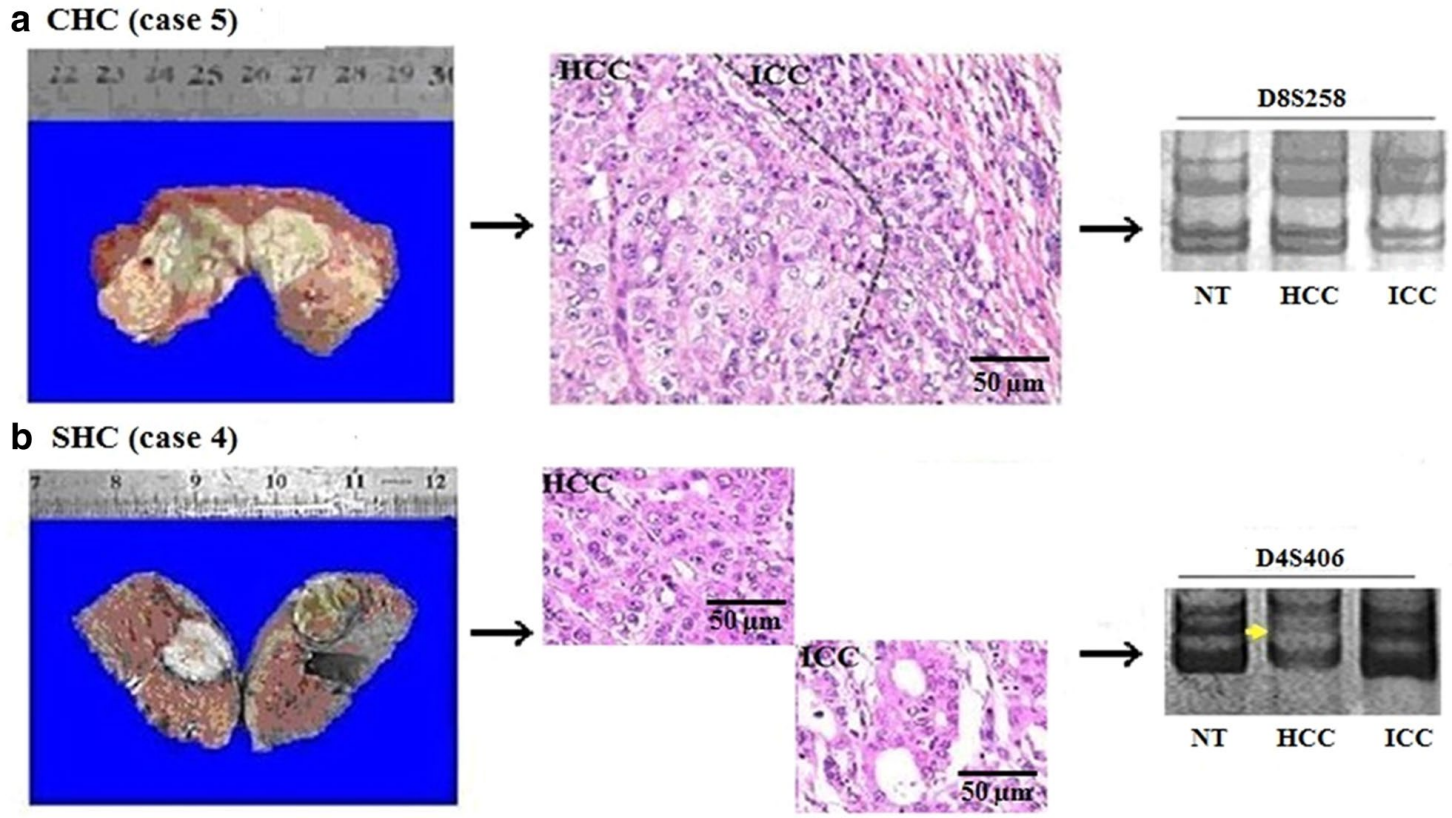
Different tumor morphology, histologic features, and LOH patterns of CHC and SHC. CHC is defined as a tumor with an intimate admixture of both HCC and ICC components, whereas SHC is defined as two separated HCC and ICC nodules arise in the same liver. Case 5 **a** shows no LOH in NT, HCC, and ICC for marker D8S258 (four *bands* presented at the same position). Case 4 **b** shows LOH in HCC for marker D4S406 (a *band* was lost in HCC compared with that in NT). *Left*, morphology; *middle*, histology; *right*, LOH pattern. *Dotted line* in **a** indicates the boundary between HCC and ICC components; *yellow arrow* in **b** indicates a reduction in density of >50% compared with paracancerous tissue. *NT* corresponding non-tumorous tissue, *CHC* combined hepatocellular and cholangiocarcinoma, *SHC* separated hepatocellular carcinoma and intrahepatic cholangiocarcinoma, *LOH* loss of heterozygosity, *HCC* hepatocellular carcinoma, *ICC* intrahepatic cholangiocarcinoma

**Fig. 2:**
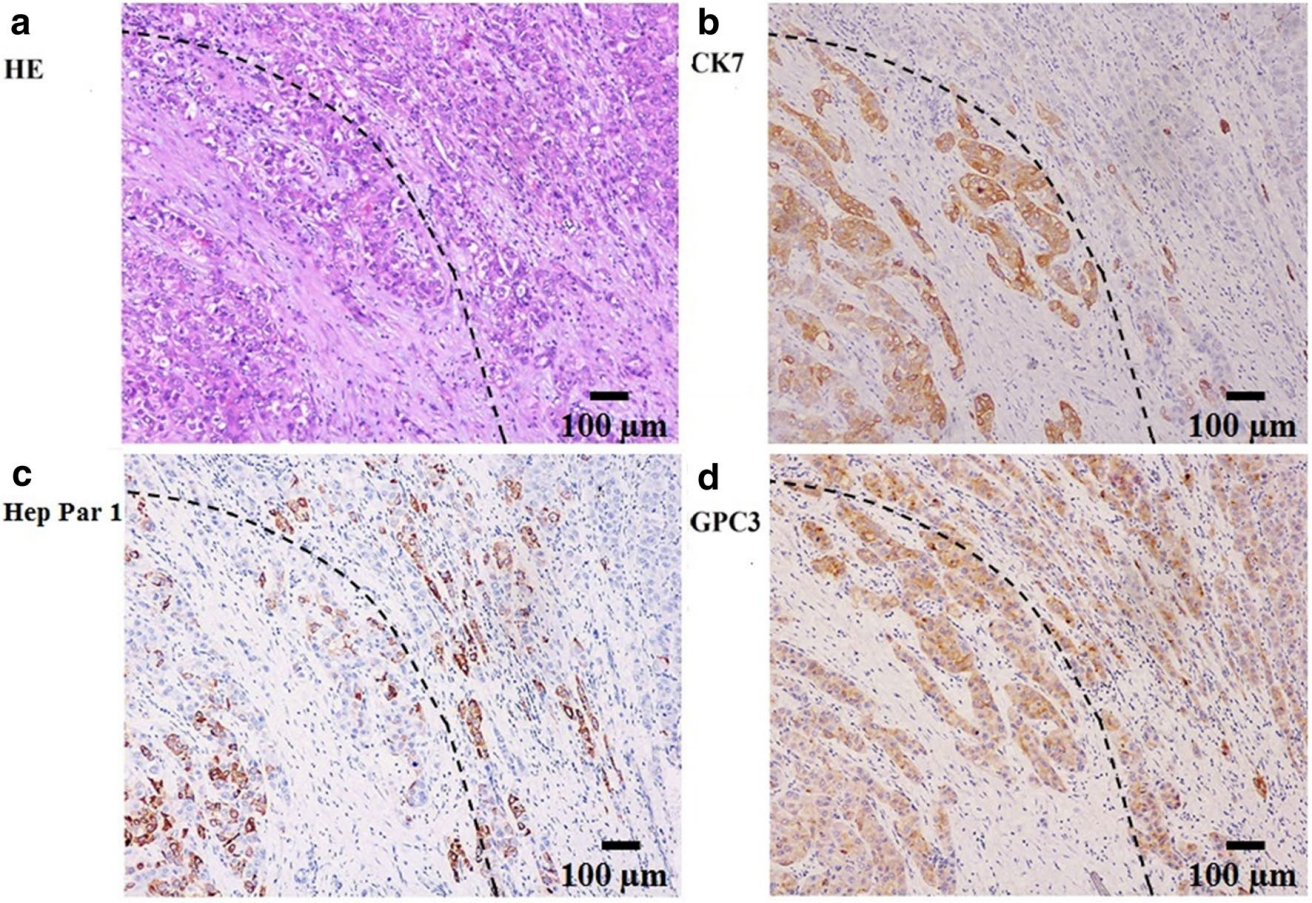
Representative HE-stained CHC sections and immunohistochemical staining for Hep Par 1, GPC3, and CK7. Dotted line indicates the boundary between HCC and ICC components. **a** Malignant glandular structures (*left part*) and solid sheets of hepatocytes (*right part*) merging into one another (HE). **b** CK7 immunostaining results in hepatocytes and biliary tissues. **c** Hep Par 1 immunostaining for hepatocytes and biliary structures. **d** GPC3 immunostaining for hepatocytes and biliary structures. *HE* hematoxylin and eosin, *Hep Par 1* hepatocyte paraffin 1, *GPC3* glypican 3, *CK7* cytokeratin 7, *HCC* hepatocellular carcinoma, *ICC* intrahepatic cholangiocarcinoma

The morphologic and histologic features of representative cases with CHC or SHC are presented in Fig. 1. CHC contained a mixture of HCC and ICC cells, whereas SHC had two separated HCC and ICC nodules in the same liver. Representative hematoxylin and eosin-stained sections and immunostaining for Hep Par1, GPC3, and CK7 are shown in Fig. 2 (the results for CK19, which are similar to those for CK7, are not presented in the figure). Positive staining for all four markers was observed in the cytoplasm of tumor cells. Of the 34 patients included in the immunohistochemical studies, 21 (61.8%) were positive for Hep Par1 expression, 32 (94.1%) were positive for GPC3 expression, 29 (85.3%) were positive for CK7 expression, and 30 (88.2%) were positive for CK19 expression. Of all 34 patients, 18 (52.9%) had co-expression of Hep Par1, GPC3, and CK7 in both the hepatic and biliary zones, 3 (8.8%) had expression of Hep Par1 (hepatocyte marker), 14 (41.2%) had expression of GPC3 (hepatocyte marker), and 11 (32.4%) had expression of CK7 (cholangiocyte marker).

### Recurrence, metastasis, and survival

**Fig. 3:**
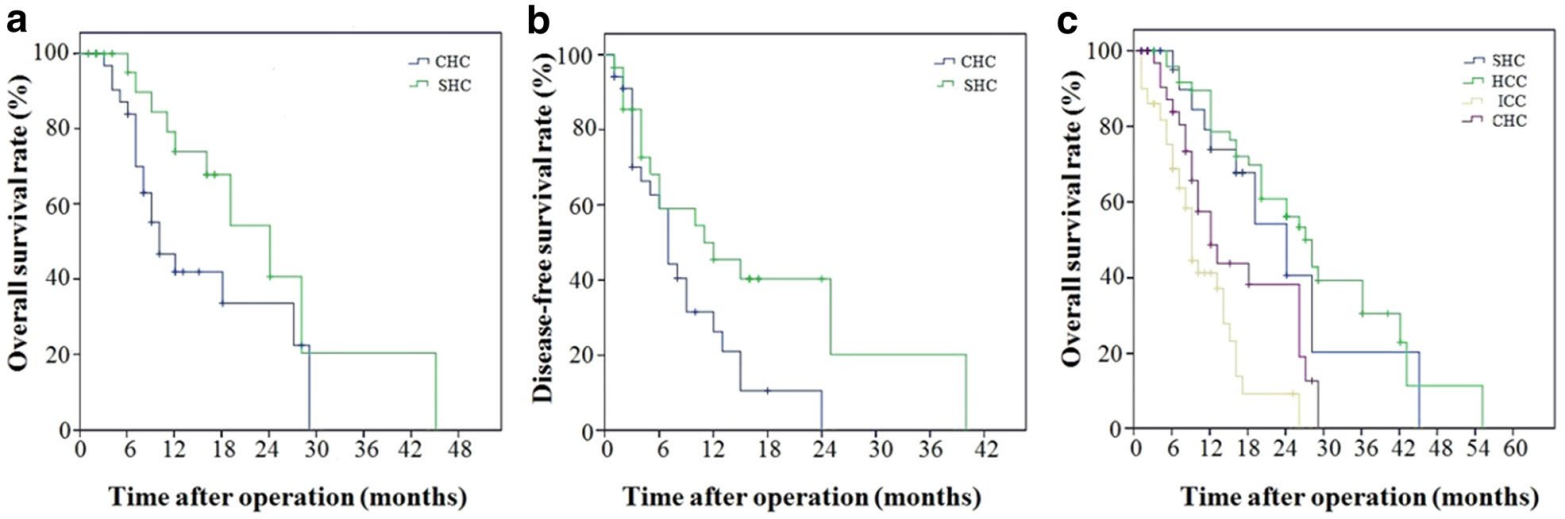
Comparison of cumulative overall and disease-free survival among patients with CHC, SHC, pure HCC, and pure ICC. **a** Cumulative overall survival curves of patients with CHC (*n* = 34) and SHC (*n* = 29). **b** Disease-free survival curves of patients with CHC (*n* = 34) and SHC (*n* = 29). **c** Cumulative overall survival curves of patients with CHC (*n* = 34), SHC (*n* = 29), pure HCC (*n* = 50), and pure ICC (*n* = 50)

Of 34 patients with CHC, 24 had tumor recurrence, with a median DFS of 7 months (range 1–24 months). After partial hepatectomy, six patients had distant metastasis, including pulmonary (*n* = 3), osseous (*n* = 1), abdominal wall (*n* = 1), and brain metastases (*n* = 1). Nineteen patients died. The median OS after partial hepatectomy was 10 months (range 3–28 months). Both the OS and DFS in the CHC group were shorter than those in the SHC group (OS: 15.4 ± 2.0 months vs. 24.0 ± 4.2 months, *P* = 0.047; DFS: 8.9 ± 1.3 months vs. 16.9 ± 3.6 months, *P* = 0.031). The OS in the CHC group was shorter than that in the pure HCC group (29.2 ± 4.1 months, *P* < 0.001) but longer than that in the pure ICC group (10.1 ± 2.3 months, *P* < 0.001) (Fig. 3).

On univariate analysis, predictive factors of OS in the CHC group were tumor size, microvascular invasion, lymph node metastasis of ICC, presence of cirrhosis, and histologic differentiation of ICC (Table 5). Multivariate analysis revealed that microvascular invasion of the HCC component, lymph node metastasis, and histologic differentiation of the ICC component were independent risk factors for OS (*P* = 0.034, *P* = 0.038, and *P* = 0.001, respectively), with hazard ratios (95% confidence intervals) of 3.293 (1.282–7.436), 3.147 (2.142–5.412), and 12.385 (3.263–14.472), respectively.

**Table 5:** Univariate analysis of factors related to survival of patients with CHC

Variable	No. of cases	Median overall survival (months)	P*
Age (years)			0.080
<50	16	12	
≥50	18	27	
Sex			0.623
Men	30	14	
Women	4	22	
Tumor size (cm)			0.012
<3	9	29	
≥3	25	10	
HBsAg			0.660
Positive	32	14	
Negative	2	6	
Microvascular invasion of HCC		1	0.019
Yes	17	0	
No	17	29	
Lymph node metastasis of ICC			<0.001
Yes	6	7	
No	28	22	
Cirrhosis			0.004
Yes	20	10	
No	14	29	
Serum AFP level (ng/mL)			
<20 vs. ≥20	10 vs. 24	18 vs. 12	0.907
<1000 vs. ≥1000	22 vs. 12	18 vs. 10	0.311
Serum CA19–9 level (ng/mL)			
<38 vs. ≥38	20 vs. 14	22 vs. 10	0.174
<100 vs. ≥100	30 vs. 4	22 vs. 14	0.958
Edmondson grade of HCC			0.847
I–II	4	8	
III–IV	30	18	
Histologic differentiation of ICC			<0.001
Well to moderate	24	27	
Poor	10	7	

*HBsAg* hepatitis B surface antigen, *AFP* ɑ-fetoprotein, *CA19-9* carbohydrate antigen 19-9, *HCC* hepatocellular carcinoma, *ICC* intrahepatic cholangiocarcinoma

^*^Log-rank test

## Discussion

CHC is a rare form of primary liver cancer, showing a mixture of hepatocellular and biliary features. In the present study, we demonstrated that the HCC and ICC components of CHC may originate from the same clone compared with SHC. CHC tended to exhibit the biological behaviors of both HCC and ICC, which may lead to poor clinical outcomes for patients with CHC.

CHC was first described by Wells in 1903 [18], and, in 1985, Goodman et al. [19] reported criteria to classify CHC into three subtypes: type I, “collision tumors,” a coincidental occurrence of HCC and ICC within the same liver; type II, “transitional tumors,” with transition from HCC differentiation to typical ICC differentiation; and type III, “fibrolamellar tumors,” a unique variant of fibrolamellar HCC. However, the World Health Organization (WHO) classification defines CHC as a tumor with an intimate and unequivocal admixture of both hepatocellular carcinoma and cholangiocarcinoma cells [3]. The WHO classification further states that CHC should be distinguished from cases of (i) SHC in which HCC and ICC arise in the same liver and (ii) collision-type tumors in which HCC and ICC are present at adjacent sites. Because there were no typical collision tumors in the present study, SHC was used as a control group for evaluating clonal differences between collision tumors and CHC.

In our study, the male-to-female ratio for CHC was 7.5:1, which is similar to the 8:1 ratio reported by Zhan et al. [20]. The frequency of HBV infection in CHC group was 94.1%, which is higher than the 65% described by Yano et al. [21] and the 75.9% described by Kim et al. [22]. However, in our study, the median age of patients with CHC was 52 years, which is younger than the median ages of 57 years and 53 years in the above reports [21, 22]. These results strongly suggest that HBV infection is a key etiological mechanism in patients with CHC, and that Chinese CHC patients may be younger at diagnosis compared with patients in the above reports [21, 22]. More interestingly, we identified a concomitant increase in AFP and CA19-9 levels in CHC patients (29.4%, 10/34). This phenomenon indicates that the HCC and ICC components of CHC have individual biological functions, with expression of both cholangiocyte and hepatocyte markers. This is an important characteristic of CHC, which may help to distinguish CHC from pure HCC or ICC before surgery.

The cellular origin of CHC is a controversial issue, but three main theories have been proposed: (i) collision (double) tumor of HCC and ICC that coincidentally exist in the same liver; (ii) subsequent differentiation of HCC or ICC into the other component; and (iii) derivation from HPCs, which have the potential to differentiate into both HCC and ICC [21, 23–25]. Despite the above speculations on the cellular origin of CHC, extensive studies on genetic alterations in CHC and comparative analyses between CHC and SHC are lacking. No previous studies have investigated the clonal origin of CHC by microsatellite LOH analysis.

As a control group of multiple clonal origins, 10 cases of SHC (20 tumor nodules) were selected as collision tumors. The differences in LOH patterns of the 10 microsatellite markers in all 10 cases of SHC were greater than 30%, which was consistent with the HCC and ICC nodules having independent origins, or a tumor type of multiple clonal origins. In comparison, the LOH incidence of 10 microsatellite markers in all 16 cases of CHC was less than 30%, suggesting that each tumor component (i.e., hepatocellular- and biliary-differentiated zones) of CHC shares the same genetic alterations and may be derived from the proliferation of a single clone. Furthermore, 52.9% of CHC had cytoplasmic co-expression of Hep Par1, GPC3, and CK7 in both hepatic zones and biliary zones, indicating that the HCC component expresses biliary markers and the ICC component expresses hepatocyte markers. This dual-modality phenotype strongly suggests that CHC originates from HPCs with the ability for multipotent differentiation.

In our study, all the metastatic lymph nodes originated from the ICC component, whereas the HCC element was mainly identified in microvascular invasion, suggesting that ICC and HCC cells from CHC maintain their distinct metastatic pathways, resulting in CHC having a dual biological mechanism associated with high malignancy. Another interesting phenomenon was the tendency for the biological behavior of CHC to be influenced by the proportion of HCC and ICC components. In the case that the HCC component was dominant, CHC was likely to exhibit the characteristics of HCC, and vice versa for a dominance of ICC components.

Because of CHC’s dual biological mechanism, patients with CHC usually have a poor prognosis. It was reported that the postoperative median survival time of patients with CHC varied from 9 to 22 months and was substantially shorter than that of HCC patients [21, 26–29]. In our study, the mean OS of patients with CHC was 15.4 months, being shorter than that of the pure HCC group (29.2 months) but longer than that of the pure ICC group (10.1 months). Multivariate analysis revealed that vascular invasion, lymph node metastasis, and histologic differentiation of the ICC component were independent risk factors for OS. These findings suggest that ICC plays a more important role than HCC in influencing the prognosis of CHC, although this requires further confirmation.

## Conclusions

In patients with CHC, the main etiological factor was HBV infection. The two components of CHC tumors (the hepatocellular- and biliary-differentiated zones) arose from a single clonal origin, which may be HPCs. CHC has a highly aggressive behavior with dual biological mechanisms.

## Abbreviations

CHC: combined hepatocellular and cholangiocarcinoma
HCC: hepatocellular carcinoma
ICC: intrahepatic cholangiocarcinoma
OS: overall survival
DFS: disease-free survival
HPCs: hepatic progenitor cells
